# Effect of Virulence Factors on the Photodynamic Inactivation of *Cryptococcus neoformans*

**DOI:** 10.1371/journal.pone.0054387

**Published:** 2013-01-18

**Authors:** Renato A. Prates, Beth Burgwyn Fuchs, Kazue Mizuno, Qurat Naqvi, Ilka T. Kato, Martha S. Ribeiro, Eleftherios Mylonakis, George P. Tegos, Michael R. Hamblin

**Affiliations:** 1 Center for Lasers and Applications, Nuclear and Energy Research Institute, São Paulo, SP, Brazil; 2 School of Dentistry, Health Department, Universidade Nove de Julho, São Paulo, SP, Brazil; 3 Division of Infectious Diseases, Massachusetts General Hospital, Boston, Massachusetts, United States of America; 4 Wellman Center of Photomedicine, Massachusetts General Hospital, Boston, Massachusetts, United States of America; 5 Department of Dermatology, Harvard Medical School, Boston, Massachusetts, United States of America; 6 Department of Pathology, University of New Mexico School of Medicine, New Mexico, United States of America; 7 Harvard-Massachusetts Institute of Technology Division of Health Sciences and Technology, Cambridge, Massachusetts, United States of America; Yonsei University, Republic of Korea

## Abstract

Opportunistic fungal pathogens may cause an array of superficial infections or serious invasive infections, especially in immunocompromised patients. *Cryptococcus neoformans* is a pathogen causing cryptococcosis in HIV/AIDS patients, but treatment is limited due to the relative lack of potent antifungal agents. Photodynamic inactivation (PDI) uses the combination of non-toxic dyes called photosensitizers and harmless visible light, which produces singlet oxygen and other reactive oxygen species that produce cell inactivation and death. We report the use of five structurally unrelated photosensitizers (methylene blue, Rose Bengal, selenium derivative of a Nile blue dye, a cationic fullerene and a conjugate between poly-L-lysine and chlorin(e6)) combined with appropriate wavelengths of light to inactivate *C. neoformans.* Mutants lacking capsule and laccase, and culture conditions that favoured melanin production were used to probe the mechanisms of PDI and the effect of virulence factors. The presence of cell wall, laccase and melanin tended to protect against PDI, but the choice of the appropriate photosensitizers and dosimetry was able to overcome this resistance.

## Introduction

Fungi are common causative agents of diseases in both immune competent as well as immune compromised patient populations. Fungal species of *Candida, Aspergillus*, and *Cryptococcus* are important opportunistic human pathogens recently and have been ranked as the seventh most common cause of infectious disease–related deaths in the United States [1]. Opportunistic fungal pathogens may cause serious superficial or invasive infections, especially in immunocompromised and debilitated patients such as those suffering from HIV infection, transplantation, corticosteroid therapy and lymphoma [2]–[5].

The incidence of invasive mycoses has increased significantly over the last three decades and now represents an exponentially growing threat for human health due to a combination of slow diagnosis and the existence of relatively few classes of available and effective antifungal drugs. For these reasons systemic fungal infections still result in high attributable mortality [2]. Cryptococcosis is an infection caused by the yeast *C. neoformans* that is unique among pathogenic fungi because it produces a polysaccharide capsule to enclose the cell. The polysaccharide capsule contributes to the overall virulence phenotype and is believed to protect against dehydration and other stress conditions [3]–[5]. The polysaccharide capsule is also released into the extracellular environment as an exopolysaccharide that has numerous toxic effects on the host innate and adaptive immune responses [6]. Cryptococcosis results from inhalation of fungal cells with subsequent lung infection and pneumonia. In the absence of an effective immune response, the fungus can disseminate to the brain to cause meningoencephalitis (the major cause of death [7]), with symptoms that include headache, fever, visual problems and an altered mental state [8]. *C. neoformans* causes an estimated 1 million cases of meningoencephalitis globally per year in patients with AIDS, leading to approximately 625,000 deaths [1]. The bulk of this disease burden is in sub-Saharan Africa, where fatal cases of cryptococcosis may exceed deaths from tuberculosis in some areas [1]. Furthermore, the infection requires prolonged antifungal therapy and it is associated with neurological sequelae and may require neurosurgical interventions [4], [9]. Cranial nerve paresthesia may occur due to fungal invasion and cranial compression secondary to cerebral edema, and paralysis can persist as permanent sequelae of the disease and involves one or more cranial nerves [6], [10]–[13].

Despite the increasing importance of opportunistic fungal pathogens, there is a limited number of effective antifungal drugs [14] and as the incidence of resistance to antifungal drugs is increasing the need for the development of new antifungal treatment modalities is pressing [15]. One potential approach under investigation is the light-based technology platform of antimicrobial photodynamic inactivation (APDI) of *C. neoformans* or other fungal pathogens [16]–[19]. APDI combines a nontoxic photoactivatable dye or photosensitizer with harmless visible light of the correct wavelength to excite the dye to the excited singlet state, which will undergo electron spin inversion to the triplet state and then generate reactive oxygen species, such as singlet oxygen and hydroxyl radicals that are toxic to cells [20]–[22]. The toxic effect of dyes and light on microorganisms was discovered accidentally more than 100 years ago employing acridine orange and eukaryotic parasites (Paramecium) [23]. Since then photodynamic therapy (PDT) has been successfully employed as a cancer modality [24] and an alternative therapeutic avenue for age-related macular degeneration [25]. The exponentially increasing threat of microbial multidrug resistance has upgraded APDI to a highly promising alternative treatment for localized infections [26], [27]. The cells that need to be killed are incubated with the photosensitizers and irradiated with light [19], [28]–[30]. The photodynamic action rapidly generates reactive oxygen species (ROS) like singlet oxygen, hydroxyl radicals, superoxide ions and lipid peroxides. Singlet oxygen has been implicated as the major causative agent of cellular damage in photodynamic process [31]. Photosensitizers are usually organic aromatic molecules with a high degree of electron delocalization that make them deeply colored [32]. Porphyrins, chlorins, bacteriochlorins, phthalocyanines as well as a plethora of dyes with different molecular frameworks have been proposed as antimicrobial photosensitizers [33], [34]. These dyes include halogenated xanthenes such as rose bengal [35], phenothiazinium dyes such as methylene blue [36], Nile blue dyes including the selenium derivative [37], cationic fullerenes (e.g. derivatives of C_60_), [38], [39] and conjugates between chlorin(e6) and poly-L-lysine [40]. The APDI platform has been proven to be effective against a range of pathogens including multidrug-resistant microorganisms [8], [41]–[45].

We have previously reported that the susceptibility of *C. neoformans* to APDI was associated with cell wall integrity [16]. The present study was aimed to extend this finding using a set of *C. neoformans* virulence-related phenotypes and characteristics. This effort was coupled with the use of a variety of unrelated photosensitizer structures that may interact with different components of *C. neoformans* cells, and produce different yields of various ROS. Moreover we assessed the uptake and localization of different photosensitizers, as well as the apoptotic mechanism of yeast inactivation.

## Materials and Methods

### Strains and Culture Conditions

The *C. neoformans* strains used in this study are listed in Table 1 [46]–[48]. Strains were grown in YPD (1% yeast extract, 2% peptone, 2% dextrose) broth for 24 h at 30°C. Cell growth was assessed with a spectrophotometer (Mini 1240, Shimadzu, Columbia, MD) at 600 nm (OD_600_). *C. neoformans* ATCC 208820 was grown in minimal medium [49] composed of 15 mM glucose, 10 mM MgSO_4_, 29.4 mM KH_2_PO_4_, 13 mM glycine, 3 µM vitamin B_1_ (pH 5.5) with and without 1.0 mM L-dopa (Sigma-Aldrich St Louis MO). L-dopa was used to induce melanin formation in the yeast. The cells were incubated for 8 days at 30°C and then APDI was performed.

**Table 1:** Description of *C. neoformans* strains used in this study.

*C. neoformans* strain (reference)	Characteristics	
KN99α [46]	Congenic ATCC 208821 (H99) MATα	Serotype A
CAP59 [47]	Congenic KN99α mating parent	Serotype A
ATCC 208819 2E-TU4 [48]	Congenic ATCC 34873 (NIH B-3501) MATα CN*lac1* Laccase negative	Serotype D
ATCC 208820 2E-TUC4 [48]	Congenic ATCC 34873 (NIH B-3501) MATα CNLAC1 Laccase positive	Serotype D

### Chemicals and Photosensitizers

**Figure 1:**
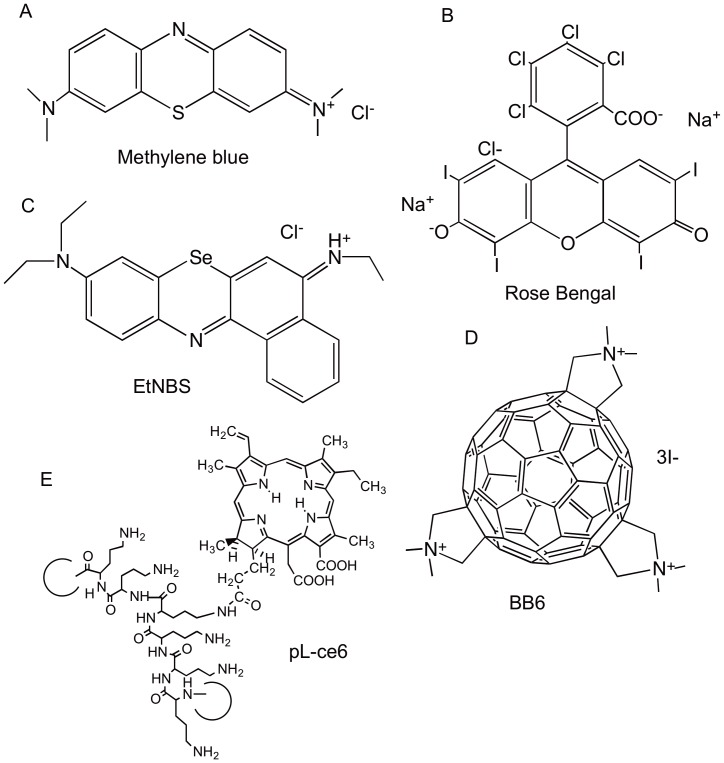
Chemical structures of the photosensitizers used in this study. (A) Methylene blue; (B) Rose Bengal; (C) Selenium Nile blue derivatrive (EtNBSe); (D) Tris-cationic fullerene (BB6); poly-L-lysine chlorin (e6) conjugate (pL-ce6).

Methylene blue as chloride salt and rose bengal were from Sigma-Aldrich (St. Louis, MO) The selenium derivative of Nile blue (EtNBSe) [37], the conjugate pL-ce6 (average 37-lysine chain) [50], and the *tris-*cationic fullerene (BB6) [39] were prepared as previously described. The chemical structures of the five photosensitizers are shown in Figure 1. Stock solutions were prepared in water (DMSO for BB6) at a concentration of 2 mM and stored for a maximum of 2 weeks at 4^o^C in the dark before use.

### APDI Studies and CFU Determination

Yeast cells were incubated with the appropriate photosensitizer dissolved in PBS at a concentration of 10 µM for 30 min in the dark. Following this period of time they were washed twice in PBS to remove the photosensitizer that was not incorporated by cells. An aliquot was taken from each sample before irradiation to quantify the number of viable cells (dark toxicity). The yeast suspensions were placed in wells of 24-well microtiter plates (Fisher Scientific) and illuminated using the halogen non-coherent lamp (LumaCare LC122, MBG Technologies, Inc., UK) with interchangeable fiber optic probes containing one of four different band pass filters. The light delivery parameters are listed in Table 2.

**Table 2:** Irradiation and fluorescence parameters for each photosensitizer.

	pL-ce6	MB	RB	EtNBSe	BB6
Wavelength (nm)	665±15 nm	665±15 nm	540±15 nm	650±15 nm	White (400–700 nm)
Total output power (mW)	700	700	450	700	1900
Fluence-rate (W/cm^2^)	40	40	30	40	112
Excitation (nm)	488	532	488	532	N/A
Emission (nm)	685	690	580	690	N/A

Yeast strains were continually irradiated from the top of the flat-bottom microtiter plate with fluences ranging from 0 to 60 J/cm^2^. During illumination, aliquots of 20 µL were taken to determine colony-forming units (CFU). The contents of the wells were constantly stirred during illumination (to ensure that yeast cells did not settle to the bottom of the wells) and mixed before sampling. The aliquots were serially diluted 10-fold in PBS to give dilutions of 10^–1^ to 10^–5^ times the original concentrations and were streaked horizontally on square YPD agar plates [51]. This allowed a maximum of seven logs of killing to be measured. Plates were incubated at 30°C for 48 h. Three types of control conditions were used: no illumination and photosensitizer, illumination in the absence of photosensitizer and incubation with photosensitizer in the dark.

### Uptake of Photosensitizers by Fungal Cells

Inocula of *C. neoformans* were incubated with photosensitizers for 30 min and the yeast cells were washed twice in PBS. One mL aliquots for each sample were centrifuged (4000×g) and the pellets suspended in 1 mL 0.1 M NaOH and 1% SDS for 24 hours. The fluorescence was measured (Spectra MAX Gemini EM, Molecular Devices, USA) at excitation and emission described in Table 3. Uptake values were calculated by dividing the number of nmol of photosensitizers in the dissolved pellet by the number of CFU obtained by a serial dilution and the number of photosensitizer molecules per cell was calculated using Avogadro’s number [16]. The excitation and emission wavelengths employed are listed in Table 2.

**Table 3:** Molecular features that could affect APDI effectiveness.

Photosensitizer	Number of +ve charges	Calc logP	Molar extinction coeff (ε) (M^−1^cm^−1^)	Type 1/Type 2
Rose Bengal	−2	3.464	89000	2 (and 1)
Methylene Blue	+1	−1.969	74000	1 (and 2)
EtNBSe	+1	5.535	55000	2 (and 1)
pL-ce6	Up to 36^a^	−2.367	40000	2
BB6	+3	1.31	50000^b^	1

a As the conjugate has 37 primary amino groups, the number of charges depends of pH and microenviroment.

b Average extinction coefficient over range 400–700-nm.

### Confocal Laser Scanning Microscopy (CLSM)

*C. neoformans* cells were incubated with the appropriate photosensitizer (10 µM methylene blue, rose bengal, EtNBSe, and pL-ce6 for 30 min) as previously described, and the cells were then labeled with MitoTracker Green™ or MitoTracker Red™ (Invitrogen, Molecular Probes, Inc., US) at 0.1 µg/mL for 5 min. BB6 lacks intrinsic fluorescence precluding the possibility of analysis by traditional fluorescence methods. Four-µL aliquots were taken from the pellet and placed on a slide and coverslip for analysis. A confocal laser microscope (Leica TCS NT, Leica Mikroskopie und System GmBH, Wetzlar, Germany) was used with excitation at 488 nm from an argon laser. Two channels collected fluorescence signals in either green range (580 nm dichroic mirror plus 525/50 nm bandpass filter) from MitoTracker Green™(Molecular Probes), and in red range (580 nm dichroic mirror plus 665 nm longpass filter) from photosensitizers. Rose bengal was observed in the green channel (580 nm dichroic mirror plus 525/50 nm bandpass filter) and fluorescence of labeled mitochondria (MitoTracker Red™, Molecular Probes) was recorded in the red channel (580 nm dichroic mirror plus 660/30 nm bandpass filter). False color images (green and red) were superimposed for the figures. Minor processing adjustments were made using Adobe Photoshop CS2.

### Apoptosis Assay

*C. neoformans* KN99α was treated with pL-ce6 mediated APDI (10 µM as described above) and then incubated with FITC-Annexin V® and propidium iodide (according to manufacturer’s instructions, Molecular Probes). Fluences of 0, 10, and 40 J/cm^2^ were delivered and then 4 µL aliquots were taken from each group following incubation and/or irradiation, and the sample was placed on a slide and coverslip for analysis. The confocal laser microscope (Leica TCS NT), with excitation at 488 nm from an argon laser was used. The cells were observed with 100× oil immersion objective and images at 512×512 pixels resolution were recorded with 0.13 µm in each side of one pixel. Two channels collected fluorescence signals in either the green range (580 nm dichroic mirror plus 525/50 nm bandpass filter) from FITC-Annexin V and in red range (580 nm dichroic mirror plus 660/30 nm bandpass filter) from propidium iodide (PI). Annexin V binds to phosphatidyl serine which is mostly localized at the inner layer of the plasma membrane in the cell without apoptotic process. However, the localization of phosphatidyl serine alters after the initiation of the apoptotic process and the phosphatidyl serine becomes exposed to the extracellular environment. Propidium iodide labels chromatin in the advanced stage of apoptosis when cell permeability has occurred.

### Statistics

Statistical analysis of the CFU data and uptake experiments data were performed using one-way analysis of variance (ANOVA). Mean comparisons were carried out with Tukey’s test with significance level at 5% (P<0.05) [52].

## Results

We employed a panel of different *C. neoformans* strains and phenotypes to explore the impact of virulence-specific characteristics and structures, (capsule, laccase enzyme and pigment production), on the APDI efficacy. Moreover, the activity of different photosensitizers is related to chemical and photochemical properties, while the efficiency to elicit antimicrobial efficacy also depends on the pathogenic cell structure and function. This was the conceptual basis for the selection and evaluation of a panel of photosensitizers with different molecular frameworks and photochemical properties. In all experiments, the number of viable cells of samples of *C. neoformans* strains treated only with laser irradiation (light alone) or with photosensitizer alone did not show a significant difference compared to the control cells (data not shown). On the other hand, there were substantial differences in the killing effect of APDI and dye uptake after incubation with the various photosensitizers. Due to the specific characteristics of each strain and the variety of hypotheses to be tested in each experiment, the data will be presented in different sections.

### Effect of Capsule on APDI

**Figure 2:**
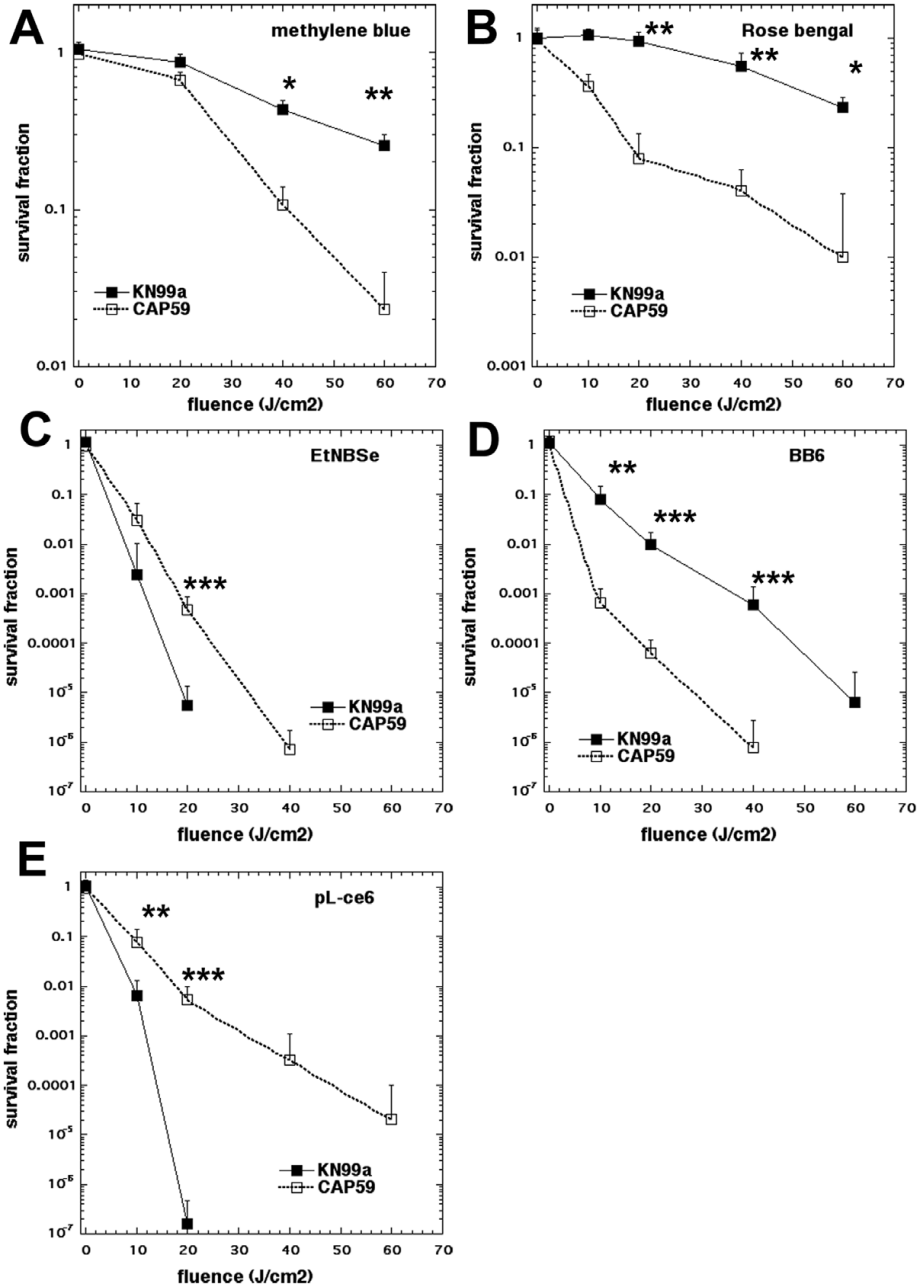
Effect of capsule on photodynamic inactivation of *C. neoformans* KN99α (black squares) and *cap59* (open squares). (A) methylene blue, (B) rose bengal, (C) EtNBSe, (D) BB6, (E) pL-ce6 were used as photosensitizers at 10 µM in PBS for 30 min followed by a wash and illumination with the wavelengths specified in Table 2. Data are means and bars are the standard deviation. * P<0.05; ** P<0.01; *** P<0.001 for survival of KN99α vs *cap59.*

*C. neoformans* has a complex outer membrane structure with the outermost layer consisting of a capsule external to the cell wall, and the cell membrane as the inner protective layer. In order to evaluate the role of capsule on APDI efficiency, we compared the killing activity of APDI mediated by different photosensitizers on two strains of *C. neoformans*: the serotype A KN99α, carrying a complete capsule, and the isogenic *cap59* mutant, with a defective capsule. Regardless of the different phototoxicity levels of each photosensitizer tested, the strain comparison clearly demonstrated the protective role of *C. neoformans* capsule in APDI. This suppressing role in phototoxicty was tightly regulated by the APDI experimental conditions (Fig. 2). This protective effect was more prominent when pL-ce6 was employed as a photosensitizer, with a difference in killing between the wild type and the isogenic *cap59* mutant strain of 5 logs and 20 J/cm^2^ (Figure 2E). Despite that, KN99α was also highly susceptible to pL-ce6-mediated APDI, showing 5 logs reduction in viability after 60 J/cm^2^. When the other photosensitizers of the panel were employed, the APDI protection was not so pronounced in the case of the capsule expressing strains. The fullerene BB6 (Figure 2D) and the Nile blue derivative EtNBSe (Figure 2C) showed the capsule provided protection of two logs of killing, while in the case of the phenothiazinium dye methylene blue (Figure 2A) and the xanthene dye rose bengal (Figure 2B) the capsule only showed about 1 log of protection. The overall efficiency of killing of the five different photosensitizers was: EtNBSe >pL-ce6> BB6> rose bengal>methylene blue.

### Effect of Laccase on APDI

**Figure 3:**
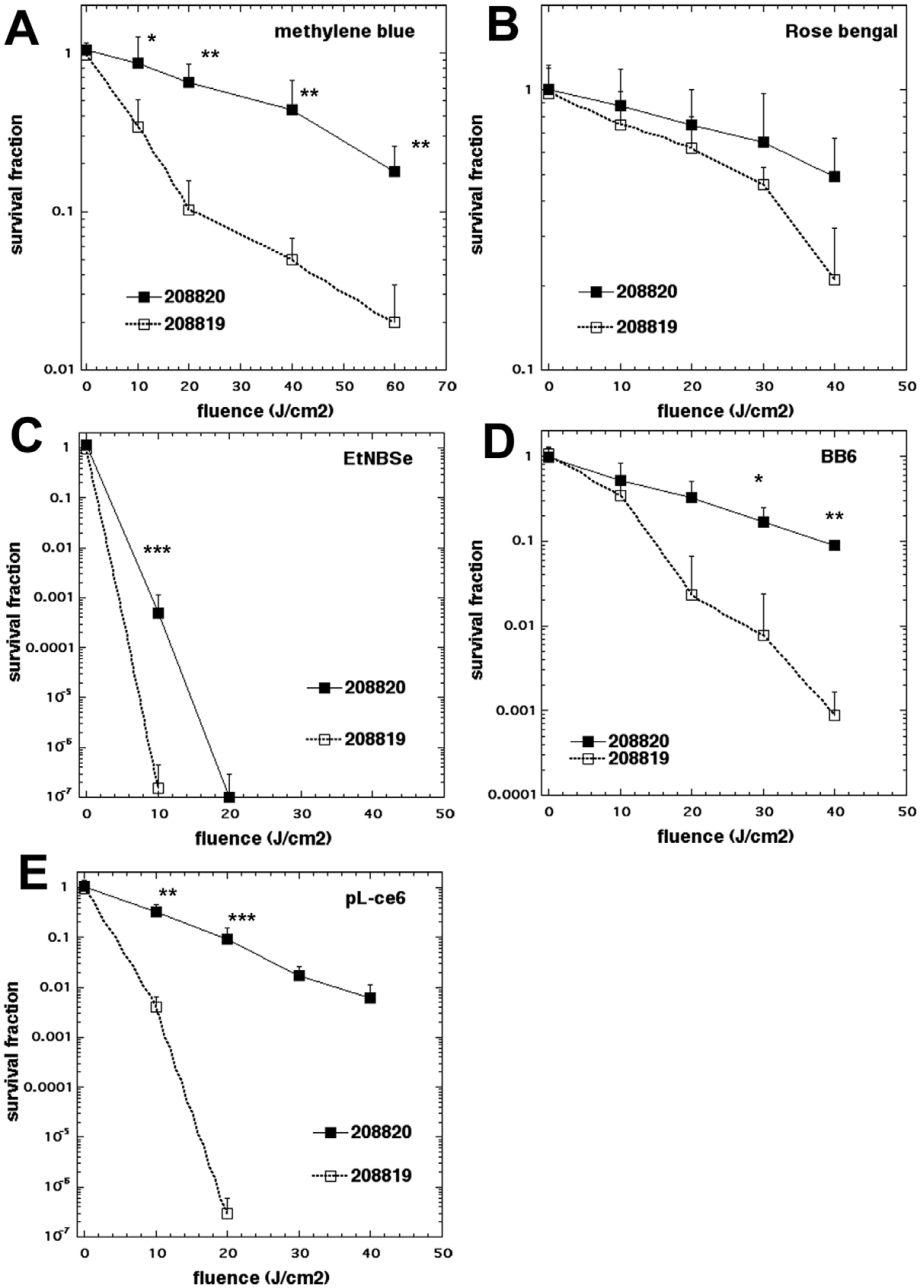
Effect of laccase enzyme on photodynamic inactivation of *C. neoformans* ATCC 208820 (laccase positive strain, black squares), and ATCC 208819 (laccase negative strain, open circles). (A) methylene blue, (B) rose bengal, (C) EtNBSe, (D) BB6, (E) pL-ce6 were used as photosensitizers at 10 µM in PBS for 30 min followed by a wash and illumination with the wavelengths specified in Table 2. Data are means and bars are the standard deviation. * P<0.05; ** P<0.01; *** P<0.001 for survival of 208820 vs 208820.

Laccase, an enzyme located at the cell wall [53] is implicated in pathogenesis of *C. neoformans* due to dopamine and non-dopamine products that confer protection against killing for this yeast through the ability to reduce reactive oxygen species and nitrogen metabolites [54]. Melanization of cryptococcal cells has been identified as a prime protective mechanism towards antifungal chemotherapy (amphotericin B, caspofungin, silver nitrate) as well as lethal doses of gamma irradiation [55]. This information prompted an evaluation of the influence of laccase production on APDI efficacy in cryptococcal cells. We compared the APDI killing effect mediated by different photosensitizers on two strains of *C. neoformans* with different degrees of melanization: *C. neoformans* 208820 is a laccase positive strain, and 208819, a laccase negative strain [55]–[57]. The laccase positive strain was less susceptible to APDI than the laccase negative strain with four out of five photosensitizers (not rose bengal) (Figure 3).

The most pronounced protection by laccase was in the case of PDI with pL-ce6 (Figure 3E), which amounted to 6 logs at 20 J/cm^2^. The next largest protection by laccase was in the case of EtNBSe (Figure 3C) where it was 3 logs at 10 J/cm^2^. APDI with BB6 showed a 2 log difference between strains at 40 J/cm^2^ (Figure 3D) and for methylene blue the difference was 1 log at 40–60 J/cm^2^ (Figure 3A). In the case of rose bengal there was no significant difference and very little killing (Figure 3B).

### Melanin Reduces Photoinactivation

**Figure 4:**
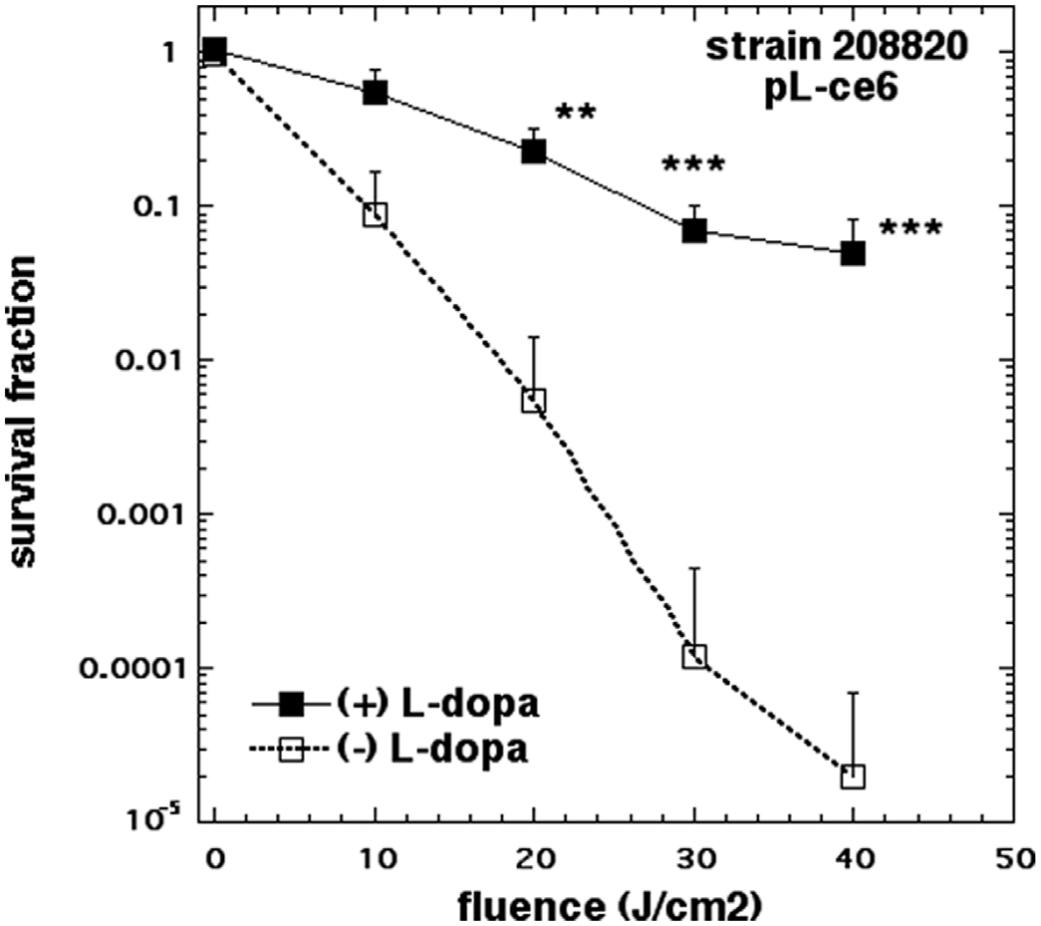
Protection of *C. neoformans* against APDI by melanin. *C. neoformans* ATCC 208820 (laccase positive strain) were grown in a minimal medium with or without L-dopa and subjected to APDI with pL-ce6 (10 µM in PBS for 30 min followed by a wash and illumination with 665-nm). Data are means and bars are the standard deviation. * P<0.05; ** P<0.01; *** P<0.001 for survival of +L-dopa vs no L-dopa.

The APDI profiling of the five photosensitizers against the two cryptococal laccase phenotypes led to the conclusion that the difference in viability reduction was the most pronounced with pL-ce6. It raised also the question regarding the degree of melanin interference with the phototoxic effect. Thus, we designed an experiment to test this hypothesis. The design involved the growth of *C. neoformans* ATCC 208820 (laccase positive strain) in minimal media, in the presence or absence of L-dopa, a catecholamine precursor required for melanin production and performing subsequent APDI with pL-ce6. There was a significant protection from killing in the cells incubated with L-dopa ranging from 1 log at 10 J/cm^2^ to 4 logs at 40/cm^2^ as shown in Figure 4. This data set confirmed that melanin production protects fungal cells from phototoxicity.

### Photosensitizer Uptake and Localization

**Figure 5:**
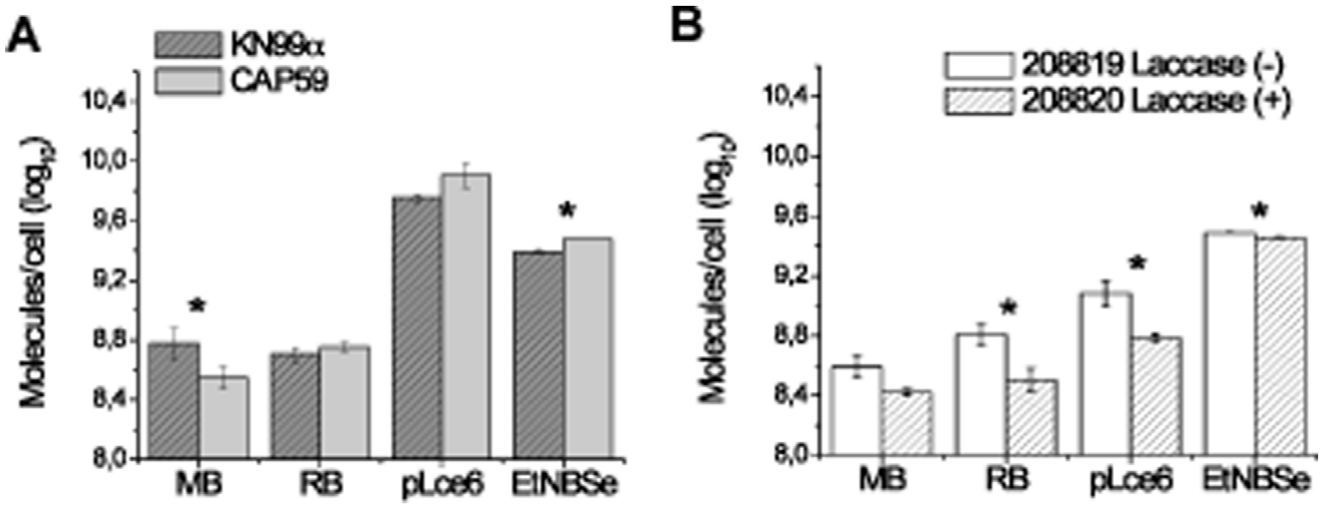
Effect of (A) capsule (KN99α and *cap59),* and (B) laccase enzyme (208820 and 208819) on photosensitize uptake. Each strain was incubated with photosensitizers at 10 µM for 30 min, as described in material and methods. Data are means and bars are the standard deviation. * p<0.05 between the positive and negative strains, for each photosensitizer.

The same set of *C. neoformans* strains as well as the photosensitizers panel used in the APDI studies (with the exception of the non-fluorescent BB6) were employed to monitor and analyze the incorporation and localization of photosensitizer inside the yeast cells. The comparison of accumulation for rose bengal and pL-ce6 in the capsule-related phenotypes KN99α and *cap59* revealed no statistical difference, between capsule positive and capsule negative strains. On the other hand, EtNBSe gave higher accumulation with by *cap59* (P<0.05), whereas methylene blue was accumulated significantly less by *cap59* compared to KN99α (P<0.05) (Fig. 5A). The uptake experiments for the laccase-related phenotypes 208819 and 208820 showed an increased fluorescence signal for all photosensitizers in the laccase deficient strain. The accumulation differences between the two strains was statistically significant for rose bengal, pL-ce6 and EtNBSe (P>0.05) (Fig. 5B).

**Figure 6:**
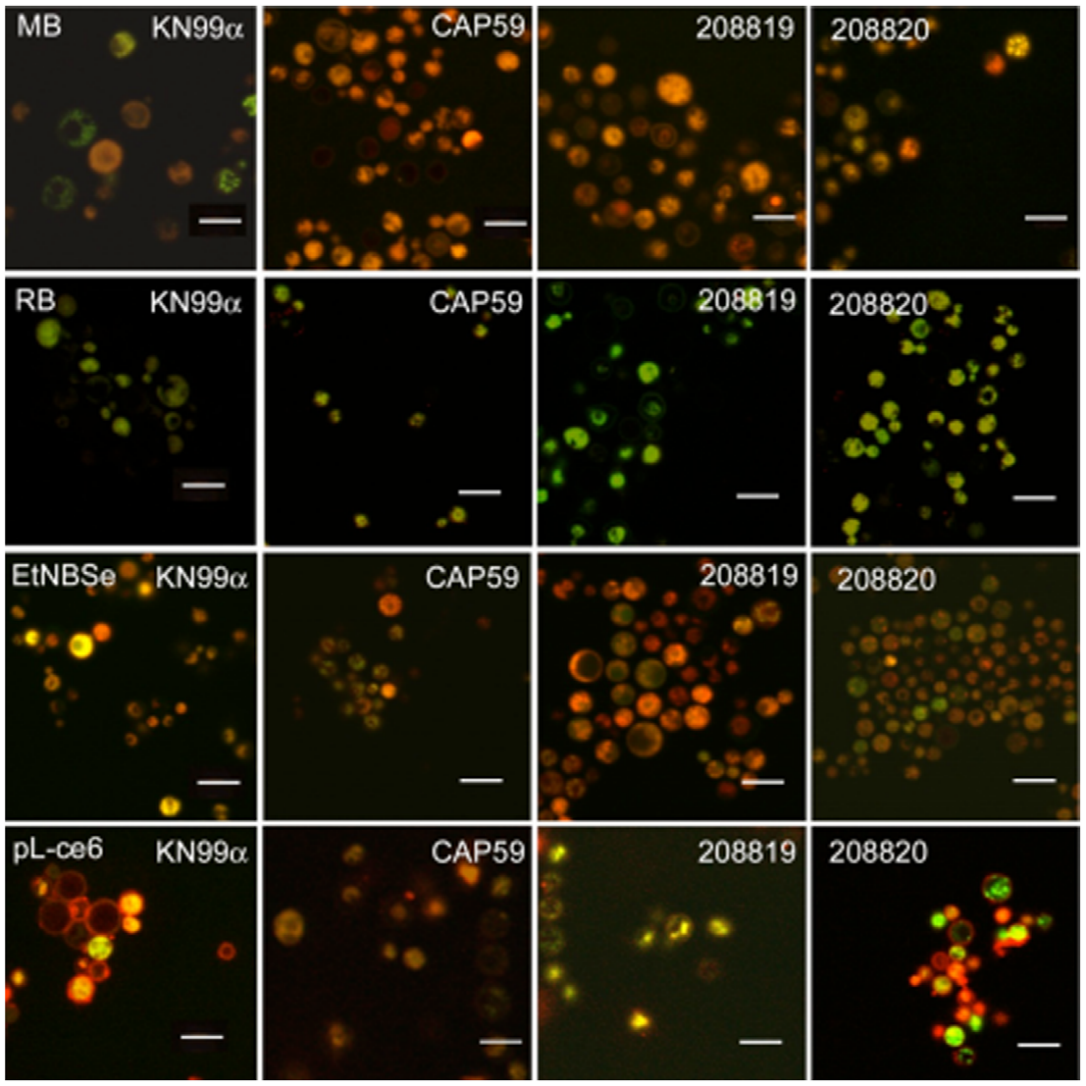
Confocal fluorescence microscopy images of *C. neoformans* strains. Cells were incubated with 10 µM photosensitizers for 30 min as described (methylene blue, rose bengal, EtNBSe, and pL-ce6) and then labeled with MitoTracker Green™ (for methylene blue, EtNBSe, and pL-ce6) or MitoTracker Red™ (for rose bengal). All photosensitizers emitted red fluorescence, except rose bengal that emits green fluorescence. We present one picture of the superimposed images for each strain incubated with each photosensitizer. Scale bars 8µm.

In order to visualize the localization of photosensitizers in the yeast cell, we conducted confocal laser scanning microscopy (CLSM) imaging employing the isogenic pairs KN99α/*cap59* and 208819/208820 incubated with members of the photosensitizers panel and Mitotracker red or green (a fluorescent probe for mitochondria). Images obtained from cells incubated with methylene blue showed intense red (methylene blue) and moderate green (Mitotracker green) fluorescence. Methylene blue was partially detected in the membrane and the nucleus of yeast cells and, the preferential site of accumulation was in the mitochondria. However, methylene blue was not only confined to this organelle, and diffuse red fluorescence was observed in the cytoplasm. Furthermore, the distribution of methylene blue in KN99α was heterogeneous and some cells just exhibited green fluorescence with no red visible (Fig. 6).

The accumulation and distribution of rose bengal (green fluorescence) appeared homogeneous in all *C. neoformans* strains. There was not a preferential site of rose bengal localization with no detectable green fluorescence in the nucleus (Fig.6).

EtNBSe exhibited an intense red fluorescence in all phenotypes with the cytoplasm and cell membrane appearing as the primary photosensitizer accumulation sites. Superimposed images of EtNBSe and Mitotracker green show photosensitizer uptake in mitochondria with no detectable red fluorescence in the yeast nuclei (Fig. 6). The CLSM images indicated external uptake of pL-ce6, which appears as red fluorescence (Fig. 6). KN99α and 208820 seems to have no internalization of pL-ce6, while the fluorescence tends to diffuse into the cytoplasm of *cap59* and 208819.

### Apoptosis Assays

**Figure 7:**
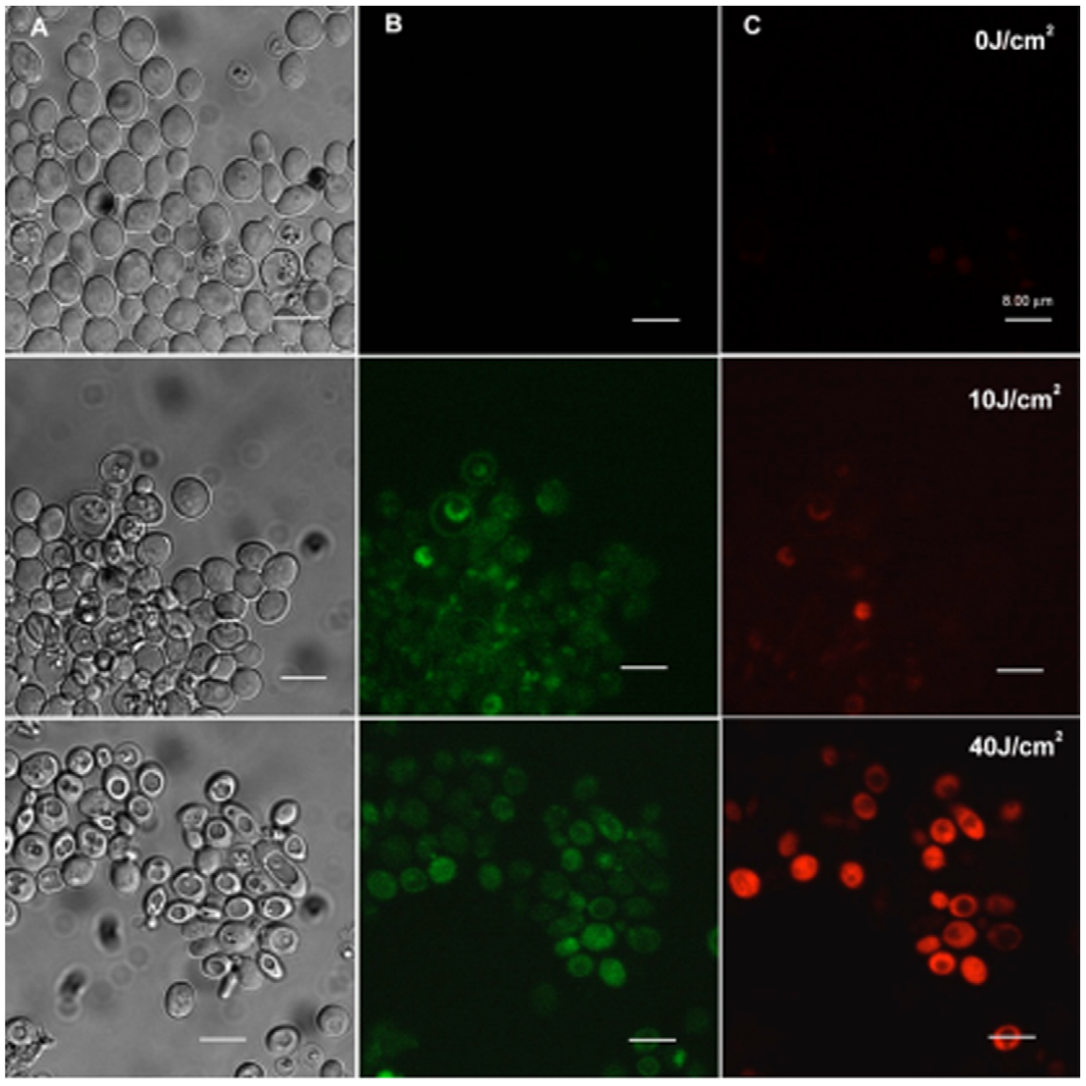
Confocal microscopy image of *C. neoformas* KN99α. Cells were treated with APDI mediated by pL-ce6 (10 µM) and then incubated with FITC-annexin V and PI. Green represents fluorescence of externalized phosphatidylserine that is correlated to the initial steps of apoptosis, and red corresponds to fluorescence of PI (advanced apoptosis/necrosis). We present three pictures of the same field: Transmittance in column A, green and red fluorescence in columns B and C respectively. The first line of figures is the stained samples before APDI (0J/cm^2^), the second line is following an irradiation of 10J/cm^2^ and the last one was irradiated with fluence of 40J/cm^2^. Scale bars 8µm.

We used an apoptotic assay developed for mammalian cells to answer the question of whether apoptosis is a significant cell death pathway in cryptococcal cells killed by APDI mediated by pL-ce6. *C. neoformans* KN99α was incubated with FITC-Anexin V® after irradiation with increasing red light fluences. When yeast cells were incubated with pL-ce6 without irradiation, their membrane showed no signs of damage or loss of integrity and fluorescence from the trackers was not detectable. Phosphatidyl serine became visible on the exterior of the yeast membrane after 10 J/cm^2^ red light. An increase in the irradiation to 40 J/cm^2^ red light led to similar levels of Annexin V (green) staining but now detectable PI fluorescence (red) was seen in the nuclei of the yeast cells. The cell modifications following PDI mediated cell death indicated the presence of an apoptotic sequence of events (Fig. 7).

## Discussion

*C. neoformans* is a pathogen that rose to prominence as the causative agent of cryptococcosis, a life-threatening disease that has emerged in parallel with the HIV/AIDS epidemic. A set of recently discovered cryptococcal pathogenesis features suggested the fungal adaptation to the mammalian environment [58]. These features include 1) remarkably sophisticated interactions with phagocytic cells to promote intracellular survival, 2) dissemination to the central nervous system, and escape [58], and 3) surprising morphological and genomic adaptations such as the formation of polyploid giant cells in the lung [59]. The recent advances in antifungal drug discovery includes only a limited number of promising countermeasures for eradication of fungal diseases including cryptococcosis [60].

The light-based technology of PDT has been suggested as a potential alternative antifungal treatments [18]. Recent conceptual and methodological studies have explored PDI in *Cryptococcus* spp. For the *C*. *neoformans* cell wall defective strain KN99α *rom2*, the photosensitizer PEI-ce6 (similar in structure to the pL-ce6 employed in the current study) exhibited an increased uptake with KN99α *rom2,* and the fluorescence was observed to penetrate the cell [16]. Rom2 is a guanyl nucleotide exchange factor in the cell wall integrity pathway, that relays the activation of the sensors by extracellular stressors through a signalling pathway that initiates the phosphorylation events of the mitogen activated protein kinase (MAPK) cascade [61], [62]. The increased uptake and the ability of the photosensitizers to penetrate and collect within the cell was likely due to defects in the cell permeability barrier that prevented the photosensitizers from being excluded. Interestingly, in this study cell death was enhanced with the addition of the antifungal caspofungin. A second study tested the effectiveness of TBO as an antimicrobial photosensitizer against *C. gattii* strains with distinct susceptibility profiles to amphotericin B and azoles. The concept of equipotent PDI between susceptible and resistant isolates was re-confirmed. A more detailed mechanistic study was attempted including determination of ROS and reactive nitrogen species (RNS, e.g. peroxynitrite) production and the catalase and peroxidase activities were measured [19].

The unique tri-layered outer structure of *C. neoformans* provides effective cell protective barriers and a challenging scenario for the penetration of potential antifungal agents including photoactive drugs. This consideration prompted a thorough investigation employing a set of both phenotypic virulence-related and a panel of photosensitizers of different structure, photophysics and photochemistry. The primary question was to shed light in the mechanistic aspects of *C. neoformans* susceptibility to PDI by studying yeast phenotypic features. A secondary aim was to identify structure-function trends and relationships between a panel of antimicrobial photosensitizers and the cryptococcal cell.

There are at least four molecular features that govern the relative effectiveness of antimicrobial photosensitizers. Firstly the number of cationic charges borne by the molecule, as it has been shown to be important to have a large number in order for photosensitizers to kill Gram-negative bacteria. Secondly is the degree of lipophilicity and molecular assymetry in the photosensitizer molecule. Although it has not been rigorously proved it is likely that this is an important consideration for anti-fungal photosensitizers. Thirdly is the molar absorption coefficient of the photosensitizer as it determines how efficiently light is harvested. Fourthly there is the ability of different photosensitizers to produce different ROS upon illumination. Singlet oxygen, hydroxyl radicals, superoxide anion and peroxides (H_2_O_2_ and lipid peroxides) have all been implicated in the antimicrobial effect of PDI. There have been several QSAR studies carried out among series of compounds of defined structure as candidate antimicrobial photosensitizer [34], [37]–[39], [63]. The following conclusions have been made. High numbers of cationic charge are important for killing Gram-negative bacteria but less so for Gram-positive bacteria and fungi. Lipophilicity is more important for killing fungi than bacteria. High molar extinction coefficients are beneficial. The ability to induce Type 1 photochemistry (hydroxyl radicals and peroxides) is important even though singlet oxygen (Type 2) remains the single most important mediator of microbial death.

In the present studies the Nile blue derivative EtNBSe was the most effective against both wild-type and mutant strains. Here the important factor may be the lipophilicity combined with the Type 1 and 2 ROS. The next most effective photosensitizer was the pL-ce6 conjugate that was particularly effective against the mutant strains. Here the factor is likely to be the large number of basic amino groups allowing binding and penetration especially in cells with defective barriers. The fullerene BB6 was next in order of effectiveness and this may be explained by the moderate lipophilicity combined with 3 cationic charges and Type 1 ROS production. Both methylene blue and rose bengal were relatively ineffective.

The killing is enhanced when the capsule and cell wall are damaged. We previously showed [16] that the addition of caspofungin, which is directed at the cell wall, enhanced killing and there was also increased cell death with the addition of fluconazole (directed at the cell membrane). These findings would suggest that the photosensitizer might work well when used in conjunction with a drug or compound that could weaken the cell wall or membrane and enhanced the effect of the photosensitizer, perhaps minimizing the amount of time that a patient would need to receive antifungal therapy. The mechanism of protection by the capsule and cell wall barriers is partly due to reduction of uptake as can be seen in Figure 5 and partly due to a restriction on diffusion of the photosensitizers inside the cells as can be seen from the confocal images in Figure 6.

How does the PDI kill the fungal cells? We showed that with pL-ce6 led to apoptosis, but it is uncertain whether this is a consequence of physical oxidative damage to components of the cell, or does the PDI generate an oxidative stressor that activates signalling pathways within cell? PEI-ce6 generates mainly singlet oxygen [64], that fungal cells do not have specific defences against. The other photosensitizer produce greater or lesser amounts of hydroxyl radicals that are more lethal than singlet oxygen on a molecule for molecule basis, but fungal cells have both constitutive [65] and inducible [66] defences against Type 1 ROS (catalase and superoxide dismutase e) that do not specifically exist against singlet oxygen. A recent report [67] showed that PDI of *C. albicans* mediated by the silicon phthalocyanine known as PC4 induced apoptosis in *C. albicans.*

Laccase is needed to produce melanin, which protects *C. neoformans* from oxidative stress. Laccase also oxidizes iron in the lungs, reducing the amount of Fe^2+^ available to alveolar macrophages that use it in oxidative processes to combat the pathogens. It should be noted that the laccase mutant and wild-type strains produced by Wiliamson and colleagues [68] belong to *C. neoformans var. neoformans* and could have some differences with *C. neoformans var. grubii* (which KN99α belongs to).

In conclusion we have shown that the capsule, expression of laccase enzyme and melanin production all protect against PDT-mediated killing of *C. neoformans.* Furthermore we have obtained information about structure-activity relationships in a panel of unrelated antifungal photosensitizers that will be valuable in ongoing efforts to rationally design high-activity photosensitizers for possible clinical application.
